# The inherent flexibility of type I non-ribosomal peptide synthetase multienzymes drives their catalytic activities

**DOI:** 10.1098/rsob.200386

**Published:** 2021-05-26

**Authors:** Sarah Bonhomme, Andréa Dessen, Pauline Macheboeuf

**Affiliations:** ^1^ Univ. Grenoble Alpes, CNRS, CEA, IBS, F-38000 Grenoble, France; ^2^ Brazilian Biosciences National Laboratory (LNBio), CNPEM, Campinas 13084-971, São Paulo, Brazil

**Keywords:** non-ribosomal peptide synthetases, flexibility, supramodular architecture

## Abstract

Non-ribosomal peptide synthetases (NRPSs) are multienzymes that produce complex natural metabolites with many applications in medicine and agriculture. They are composed of numerous catalytic domains that elongate and chemically modify amino acid substrates or derivatives and of non-catalytic carrier protein domains that can tether and shuttle the growing products to the different catalytic domains. The intrinsic flexibility of NRPSs permits conformational rearrangements that are required to allow interactions between catalytic and carrier protein domains. Their large size coupled to this flexibility renders these multi-domain proteins very challenging for structural characterization. Here, we summarize recent studies that offer structural views of multi-domain NRPSs in various catalytically relevant conformations, thus providing an increased comprehension of their catalytic cycle. A better structural understanding of these multienzymes provides novel perspectives for their re-engineering to synthesize new bioactive metabolites.

## Graphical abstract

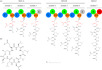

## 1. Introduction: the inherent flexibility of non-ribosomal peptide synthetases

**Figure 1.**
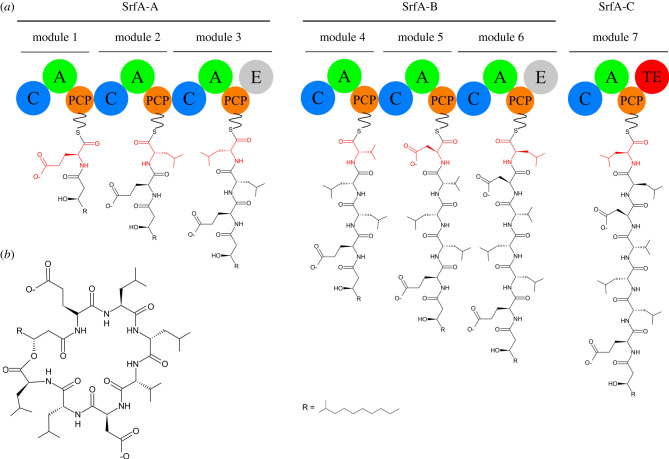
Organization of the surfactin NRPS. (*a*) The surfactin NRPS is composed of three polypeptides: SrfA-A, SrfA-B and SrfA-C. Each polypeptide is composed of one or several modules, each one being responsible for the incorporation of an amino acid, highlighted in red in the growing metabolite. Module 1 is the initiation module, module 7 is the termination module and those in between are elongation modules. The addition of an amino acid into the metabolite requires the cooperation between domains, represented as coloured spheres. All surfactin NRPS modules possess a unique non-catalytic domain, the PCP (in orange) that tethers the growing metabolite. Each module also contains at least two catalytic domains: a condensation domain (C, in blue) and an adenylation domain (A, in green). Additionally, modules 3 and 6 possess an optional epimerization domain (E, in grey). Finally, the termination module ends with a thioesterase (TE, in red) domain that releases and cyclizes the surfactin molecule. (*b*) Chemical structure of the surfactin.

Natural products are secondary metabolites synthesized by microorganisms in order to adapt to their environment [1]. Many of these natural products have been used for medical purposes, such as the antibiotics daptomycin and vancomycin [2] and the anti-cancer molecule bleomycin [3]. Among these natural products, non-ribosomal peptides (NRPs) make up a vast class of peptide-based metabolites, synthesized independently from the ribosome by large machineries named non-ribosomal peptide synthetases (NRPSs). For instance, the surfactin lipopeptide is synthesized by the 1 MDa surfactin NRPS from *Bacillus subtilis* (figure 1) [4]. Recent progress in whole genome sequencing has revealed the existence of numerous NRPS gene clusters among bacteria and fungi, mostly of unknown function [5–7]. Nevertheless, the structural understanding of the machineries that produce these metabolites has long remained limited to studies of isolated domains [8] until relatively recently, and has evolved dramatically in the last few years.

NRPSs are classified into two categories, type I and type II. In type II NRPSs, the incorporation of an amino acid into the metabolite necessitates the involvement of several domains carried by distinct proteins [9]. By contrast, the type I NRPS megaenzymes use an assembly line strategy (figure 1) with modules that act sequentially, each being responsible for the incorporation of an amino acid into the final metabolite. A NRPS assembly line is composed of an initiation module (module 1 in figure 1*a*), a termination module (module 7 in figure 1*a*) and one or several elongation modules (2 to 6 in figure 1*a*). In general, several modules are fused into a single polypeptide. Polyketide synthases (PKSs) that are also modular megaenzymes, adopt the same assembly line logic as NRPSs but they use small carbon chain substrates instead of amino acid substrates [10]. This similar strategy explains the existence of numerous hybrid NRPS/PKS assembly lines that produce hybrid peptide-polyketide metabolites [11].

Most modules are composed of two catalytic domains, the adenylation (A) and the condensation (C) domains (figure 1*a*); each NRPS module also incorporates the non-catalytic, but essential, peptidyl carrier protein (PCP) domain [12]. In this review, we will consider that a classical module starts with a C domain and ends with a PCP domain (figure 1). The PCP domain functions as an anchoring platform for shuttling substrates to the different catalytic domains within a module; it also allows the transport of the modified substrate from the upstream to the downstream module (figure 1*a*). PCP domains, whose masses are in the range of 10 kDa, have been mostly studied in isolated form by NMR [13–15]. They fold as a right-handed four helix bundle, with the four helices (I, II, III and IV) being connected by loops. At the N-terminus of helix II, all PCP domains possess a conserved serine residue that serves as an attachment point for a phosphopantetheine arm (PPant arm). This post translational modification is catalysed by phosphopantetheinyl transferases (PPtases) that convert apo-PCPs into holo-PCPs [16]. The 20 Å long PPant arm displays a free thiol at its extremity that allows the loading of various substrates. In this review, the term ‘loaded-PCP’ will be used to refer to PCPs modified with a PPant arm and loaded with a substrate. Indeed, a number of NRPSs have been structurally characterized in various productive conformations by employing a promiscuous PPtase, such as Sfp from *Bacillus subtilis* [17], for the loading of substrates, mimics or dead-end inhibitors, onto the megaenzymes [18–22].

The PCP domain delivers substrates to the catalytic domains. First, the adenylation (A) domain activates the incoming acid monomer. A domains select very diverse monomers including α-L- or α-D-amino acids, β-amino acids or aryl acids [23]. Subsequently, the condensation (C) domain catalyses peptide bond formation between two PCP-tethered monomers. Many optional tailoring domains that further chemically modify the metabolite under construction can also be found in NRPSs [24]. For example, the epimerization (E) domain converts natural L-amino acids into D-amino acids (modules 3 and 6 in figure 1*a*). Finally, each assembly line ends with a domain, such as a thioesterase (TE) or a reductase (Re) (module 7 in figure 1*a*), that releases the final product. This final stage can introduce further diversity in the peptide as the release can occur either by hydrolysis or cyclization. Interestingly, since surfactin release occurs via macrolactonization [25,26], surfactin metabolites contain both amide and ester bonds, thus deserving the designation of ‘depsipeptide’ (figure 1*b*) [27].

NRPS flexibility allows the conformational changes required for the interactions between the PCP and the catalytic domains. However, this flexibility is a drawback to structural characterizations of large NRPS fragments and, accordingly, successful structural studies have often required the employment of chemical tools to reduce conformational heterogeneity [19–22]. Nevertheless, characterizing the movements of NRPS multienzymes is a requirement for the detailed understanding of these fascinating machineries. In this review, we focus on recent aspects of NRPS flexibility that allow PCP movements during a catalytic cycle, by describing both the successive conformations adopted by these enzymes during a cycle as well as movements that engender passage from one conformation to the next.

## 2. Known non-ribosomal peptide synthetase structures

**Figure 2.**
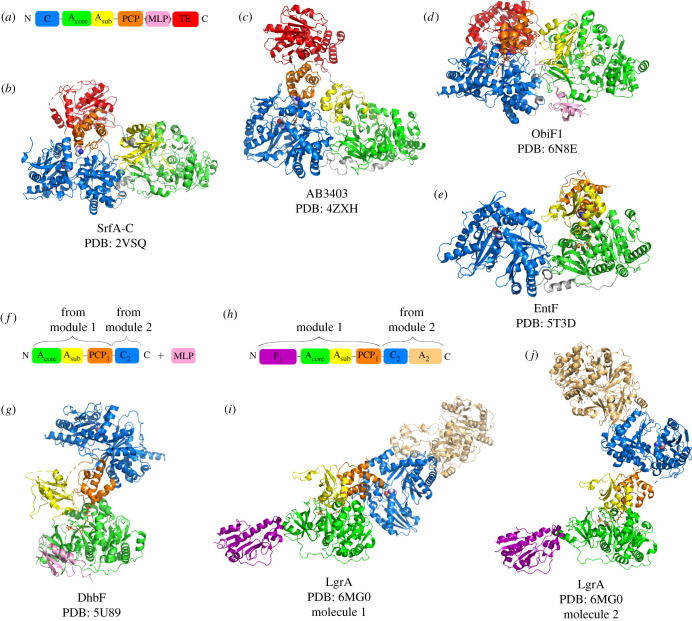
Structural gallery of NRPS modules. If present, the PPant arm attached to the PCP domain is represented as sticks. (*a*,*b*) Domain organization and crystal structures of the termination modules SrfA-C (PDB code: 2VSQ) [19], (*c*) AB-3403 (PDB code: 4ZXH) [21], (*d*) ObiF1 (PDB code: 6N8E) [22] and (*e*) EntF (PDB code: 5T3D) [21]. In addition to the PCP domain, these modules are composed of condensation (*c*), adenylation (*a*) and thioesterase (TE) domains. No electron density was detected for the TE domain of EntF. The catalytic His of the C domain is represented in spheres. (*f*,*g*) Domain organization and crystal structure of DhbF, a cross-module construct (PDB code: 5U89) [16]. (*h–j*) Domain organization and crystal structures of a five-domain construct of LgrA (PDB code: 6MG0) [24]. LgrA starts with a formylation (*f*) domain. The domain in pink in ObiF1 (*d*) and DhbF (*g*) represents MLP, an A domain activator.

**Figure 3.**
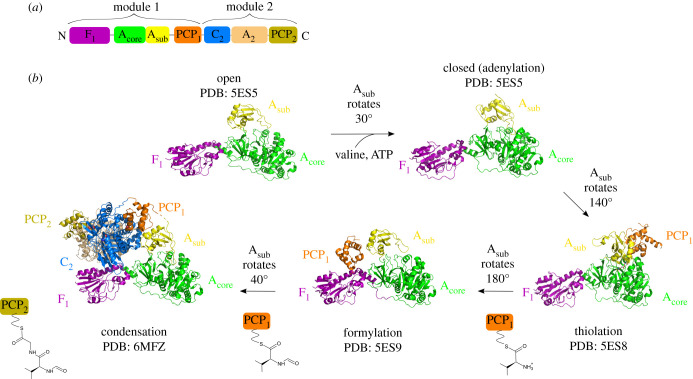
Catalytic cycle of LgrA, a dimodular NRPS. (*a*) Domain organization of LgrA. For clarity, the inactive epimerization domain of LgrA, that follows the PCP_2_ domain, is not shown. (*b*) Catalytic cycle of LgrA illustrated by five crystal structures (PDB codes: 5ES5, 5ES8, 5ES9 and 6MFZ) [23,24]. Four different structures of module 1 reveal details about the catalytic cycle of an initiation module. The PCP_1_ domain is disordered in the open and closed states. First, the open state allows binding of valine and ATP. The closed state is the conformation which is relevant for the activation of valine by ATP (i.e. the adenylation state). The thiolation conformation captures the transfer of valine from the A_1_ domain to the PPant arm of the PCP_1_ domain. In the formylation conformation, the F_1_ domain adds a formyl group to valine, still attached to the PCP_1_ domain. The structure of the full dimodular NRPS allows the visualization of the condensation conformation. After condensation, Gly activated by module 2 is covalently bound to formyl-Val by a peptide bond. In all presented structures, the F_1_-A_core_ bidomain adopts similar conformations whereas the A_sub_ subdomain and the PCP_1_ domain are positioned differently. Rotations of the A_sub_ subdomain induce movements of the PCP_1_ domain due to the linker between them.

The field of NRPS structural biology achieved major breakthroughs in the last five years with the publication of several crystal structures of multi-domain NRPSs, largely due to the work of the Schmeing and Gulick groups (figure 2) [19–22,28,29]. These results add to a high number of structures of individual catalytic domains and PCP-containing didomains, solved by NMR, X-ray crystallography or a combination of both techniques [8]. Each domain adopts the same fold in different structures, independent of the number of domains present in the protein. Nevertheless, understanding the organization of full modules as well as module–module interactions is essential to provide a better insight into these assembly lines. For a long time, however, the intrinsic flexibility of NRPSs prevented the structural characterization of full modules at high resolution. The first structure of a full module, that of the termination module of surfactin, SrfA-C, was solved by X-ray crystallography in 2008 (figures 1*a* and 2*a*,*b*) [30]. Since then, crystal structures of three other termination modules have been solved: AB3403 from an uncharacterized pathway of *Acinetobacter baumanii*, the enterobactin termination module EntF and ObiF1 from the obafluorin assembly line (figure 2*c–e*) [20,29,30]. In 2017, a combined effort in X-ray crystallography and negative staining electron microscopy (EM) provided insights into the structures of the last two modules of DhbF involved in the synthesis of bacillibactin (figure 2*f,g*) [21]. Lastly, the structural elucidation of the dimodular protein LgrA, from the gramicidin synthetase complex that contains an initiation and an elongation module, was a breakthrough in the comprehension of supramodular NRPS organization. Indeed, 12 crystal structures of LgrA fragments complexed with substrates, substrate analogues and dead-end inhibitors were solved and provided a full picture of the catalytic cycle of a dimodular NRPS (figures 2*h*–*j* and 3) [19,22].

## 3. Loading of the amino acid onto the peptidyl carrier protein domain

The adenylation (A) domain is divided into two subdomains: the N-terminal subdomain, A_core_, consists of around 400 amino acids, while the C-terminal subdomain, A_sub_, comprises around 100 amino acids [31]. The A domain catalyses two reactions: the activation of the acid monomer using ATP (adenylation) and its subsequent transfer to the PCP domain (thiolation or thioesterification). The A domain is able to adopt several conformations that have been described as ‘the domain alternation cycle’ and which are supported by several structures of complete NRPS modules (figure 3) [19,20,22,31]. Remarkably, the thiolation state has been characterized multiple times using non-hydrolysable analogues (figure 3*b*, thiolation conformation) [18–20,22]. For example, the structure of PA1221, a natural A-PCP didomain NRPS from *Pseudomonas aeruginosa*, was obtained both in its apo form and in a loaded form locked in the thiolation conformation through the use of the inhibitor valyl-adenosine vinylsulfonamide (AVS) [32]. In the apo form, the electron density for the PCP domain was absent, suggesting the domain was flexible, whereas the whole didomain was visible in the presence of the AVS inhibitor, indicating that it stabilized the A-PCP interface. This suggests that the numerous crystal structures of NRPSs in the thiolation conformation do not reflect a preferentially adopted conformation *in vivo* but, more likely, a conformation that favours crystallization.

The cycle starts when the A domain, in an open conformation, is available for substrate binding (figure 3*b*, open conformation) [31]. The A_core_ subdomain contains the monomer binding pocket that accommodates ATP and the acid monomer. Upon substrate binding, a 30° rotation of the A_sub_ subdomain leads to the closed conformation, which is then suitable for adenylation (figure 3*b*, closed conformation) [19]. This conformation allows the entry of an A_sub_ loop into the A_core_ subdomain; this loop contains a conserved catalytic lysine that stabilizes the acid substrate and ATP [33]. Subsequently, after adenylation and pyrophosphate release, a 140° rotation of the A_sub_ subdomain allows the conversion between the closed and the thiolation conformations, the latter being able to catalyse thioesterification (figure 3*b*, thiolation conformation) [31].

The rotations of the A_sub_ subdomain are facilitated by a flexible hinge region containing a conserved aspartic acid or a lysine, located in the A_core_-A_sub_ linker [34]. The structure of an adenylate-forming enzyme with the hinge residue mutated into proline revealed an enzyme blocked in the adenylation conformation. Consistent with the structure, the mutant enzyme was still capable of adenylation, but not of thiolation [34]. The importance of the hinge residue has also been demonstrated in the context of the multi-domain NRPS EntF, in which the same hinge residue mutation abolished enterobactin production [35]. Therefore, the flexibility of the hinge residue in the A_core_-A_sub_ linker is essential to allow the movement of the A_sub_ subdomain relative to the A_core_, which is necessary to allow conformational changes of the whole module. Indeed, rotations of the A_sub_ subdomain drive movement of the PCP owing to the linker connecting the two domains. Analysis of the A-PCP linker region reveals that it contains multiple prolines, absent in standalone A domains [35]. These prolines might rigidify the A-PCP linker, thus facilitating movements of the PCP domain in concert with the movements of the A_sub_ subdomain [35].

## 4. Modification of the peptidyl carrier protein-tethered amino acid

The vast diversity of NRPs arises in part from the action of tailoring domains, such as cyclization (Cy), epimerization (E), formylation (F), ketoreductase (KR), methyltransferase (Met) and oxidase (Ox) domains, that modify the peptide under construction [19,36–40]. It is not unusual for tailoring domains to be inserted within A domains, that are then called interrupted A domains [39,41]. The structure of LgrA in the formylation conformation provides insight into the mechanism of amino acid modification by a tailoring domain, in this case the formylation of PCP-bound L-valine by an F domain using a formyltetrahydrofolate cofactor (figure 3*b*, formylation conformation) [19]. Since the F and PCP domains are separated by the large A domain (figure 3*a*), a substantial conformational change must occur to allow the PCP domain to position its PPant arm in the F active site. The A_sub_ subdomain rotates 180° from its position in the thiolation conformation while the PCP domain rotates 75°, thus moving 60 Å away from its thiolation position (compare figure 3*b*, thiolation and formylation conformations). The interaction surface between the F and PCP domains is very limited. Unfortunately, there is no structure of the full dimodular LgrA in the formylation conformation. It is worth noting that the structure of dimodular LgrA (figure 3*b*, condensation conformation) reveals that the position occupied by the PCP_1_ domain in the formylation conformation is occupied by the C domain at a later stage of the catalytic cycle [22].

## 5. Elongation of the donor peptide chain with an acceptor amino acid

Elongation is the only reaction that necessitates interactions between domains belonging to different modules. This reaction is catalysed by a condensation (C) domain or, more rarely, by a cyclization (Cy) also called heterocyclization (HC) domain, that catalyses cyclization after condensation [42]. If the two modules belong to the same polypeptide, PCP and C domains are directly connected by a linker (figure 3*b*, condensation conformation). If they belong to different polypeptides, docking domains facilitate the interaction between the upstream PCP and the downstream C domain [43]. The C domain of an elongation module *n* catalyses peptide bond formation between the growing chain carried by the upstream PCP domain (donor PCP), that belongs to module *n-*1, and the activated amino acid carried by the downstream PCP domain (acceptor PCP) located on the same module *n* (figure 3*b*, condensation conformation) [42]. The growing peptide chain is directly transferred from one PCP domain to the next, without attachment to the C domain. Thus, the two PCP domains must bind simultaneously to two different binding sites on the C domain; these are referred to as donor and acceptor binding sites. However, the C domain must discriminate between the two PCP domains to maintain the directionality of the assembly line. Therefore, the condensation reaction results from the interaction between three domains (the C, the donor PCP and the acceptor PCP domains).

The C domain adopts the V-shaped pseudo-dimeric fold seen in the chloramphenicol acyltransferase family [42]. It is divided into N-terminal and C-terminal lobes, and the active site is located inside the N-terminal lobe, at the centre of a tunnel formed by the interface between the two lobes (figure 2). The structures of three termination modules, SrfA-C, AB3403 and ObiF1, show the acceptor PCP domain docked onto the C domain acceptor site (figure 2*a*–*d*) [20,29,30]. In SrfA-C, although the PCP domain is in its apo form due to a Ser-Ala mutation, the Ala is located 16 Å away from the catalytic His of the C domain, suggesting that this structure is compatible with a productive condensation reaction (figure 2*b*) [30]. Both holo-AB3403 and holo-ObiF1 show the PCP domain docked onto the C acceptor site with the PPant arm inserted through a tunnel that allows the positioning of the final thiol in proximity to the catalytic His of the C domain (figure 2*c*,*d*) [20,30]. It is worth noting that, as opposed to what was observed for the PCP interaction with the A domain, no substrate or inhibitor was needed to favour the interaction between the acceptor PCP and C domains.

Several dimodular LgrA structures have revealed for the first time the productive interaction between a donor PCP domain and its corresponding C domain (figure 3*b*, condensation conformation) [22]. Indeed, in four structures of LgrA, the donor PCP_1_ domain is docked in the C_2_ donor site, presenting its conserved Ser towards the catalytic His of the C domain, the two residues being separated by less than 20 Å. The donor PCP_1_ domain globally has the same orientation in these structures and three of them show electron density for the PPant arm in the donor tunnel of the C domain. However, the structure of F_1_-A_1_-PCP_1_-C_2_ with f-Val loaded onto the PPant arm of PCP_1_ reveals that the reactive thioester group must slightly modify its position in order to be properly positioned for attack by the acceptor amino acid. The authors hypothesized that the donor substrate could only be correctly positioned in the presence of the PCP-bound-acceptor substrate in the active site of the C domain [22]. The importance of the loading status of the carrier protein domain for megaenzyme conformation has already been demonstrated for the modular PKS megaenzymes that incorporate domains analogous to the C, A and PCP of the NRPS systems [44,45]. Indeed, structures of the PikAIII PKS module loaded with various substrates obtained by cryo-electron microscopy revealed that the acyl carrier protein (ACP) domain adopts dramatically different positions according to the nature of the substrate loaded onto the ACP.

A detailed comprehension of the condensation reaction requires structural information on a NRPS including at least a donor PCP, an acceptor PCP and a condensation domain. The structure of holo-LgrA F_1_-A_1_-PCP_1_-C_2_-A_2_-PCP_2_ reveals both donor and acceptor PCPs docked onto a single C domain (figure 3*b*, condensation conformation) [22]. 29 Å separate the two PCP Ser residues loaded with their 20 Å-long PPant arms. The Ser from the acceptor PCP_2_ is located 15 Å away from the catalytic His of the C domain while the Ser from the donor PCP_1_ is 18 Å away from it. These distances are compatible with the proximity between substrates required for nucleophilic attack. Unfortunately, there is no electron density for the PCP PPant arms so the detailed interaction of donor and acceptor substrates cannot be deduced from this structure.

The detailed view of PCP-bound-substrates in the condensation conformation can be obtained using a mechanism-based probe, recently designed by the Gulick and Aldrich groups [46]. Although the structure of LgrA F_1_-A_1_-PCP_1_-C_2_-A_2_-PCP_2_ shows that an inhibitor is not necessary to lock a dimodular NRPS in the condensation conformation, this new chemical probe stabilized the interaction between the donor PCP, the acceptor PCP and the C domains. The enterobactin assembly line served as a model to prove the functionality of this probe [46]. The authors were able to mimic the PPant arm loaded with the natural substrate of the donor PCP by replacing the whole acyl-thioester portion of the substrate by a non-hydrolysable analogue incorporating a ketone functionality, thus preventing the release of the loaded substrate. The resulting crypto-PCP was shown to bind to the donor site of the C domain and these results allowed the construction of a model where the crypto-PCP inserts its unnatural PPant arm into the donor tunnel. The authors assumed that the pantetheine probe would then react with the natural acceptor substrate loaded on the acceptor PCP, forming an imine bond instead of the natural peptide bond formed between substrates. In this configuration, both PCP domains should be docked onto the C domain and linked together via their PPant arms connected through an imine bond. Therefore, this probe should help stabilize the interaction between the C and PCP domains during condensation and could lead to more crystal structures of bimodular NRPS locked in the condensation conformation.

The structure of the LgrA PCP_1_ domain has been solved in association with its three catalytic partners (A_1_, F_1_ and C_2_ domains, figure 3*b*); therefore, the comparison of the three crystal structures provides insights into the conformational changes that allow the PCP_1_ domain to shuttle between its three partners [19,22]. As described above, the large movements required for the PCP domain to reach its different catalytic partners are mainly driven by conformational changes of the A_sub_ subdomain that are transferred to the PCP domain by the A_sub_-PCP rigid linker. Shifting from the formylation to the condensation conformation, the PCP_1_ domain must cross 30 Å, achieved through a rotation of 40° of the A_sub_ subdomain (compare figure 3*b*, formylation and condensation conformations). Similarly, after condensation, the PCP_1_ domain must detach from the C domain and travel back 50 Å to return to the A_1_ active site (compare figure 3*b*, condensation and thiolation conformations), achieved by a rotation of 150° of the A_sub_ subdomain. Even in the absence of structures that show the second LgrA module in the thiolation conformation, we can easily extrapolate that the movements seen in module 1 could be similar in module 2.

Interestingly, a NRPS module can start a second catalytic cycle before the first one is complete [20]. For example, the structure of LgrA in the condensation conformation (figure 3*b*) shows the PCP_1_ domain in the peptide donation conformation while the A_1_ domain is in the closed conformation and can thus catalyse adenylation [22]. After adenylation, the aminoacyl-AMP is tightly sequestered in the A domain active site in the absence of the available PCP [47,48]. Subsequently, the A_1_ domain can catalyse thiolation as soon as the C_2_ domain has catalysed condensation which will liberate the PCP_1_ domain. This decoupling between different domain activities likely increases the synthesis rate of NRPSs.

## 6. Release of the peptidyl carrier protein-tethered peptide

The structures of four termination modules harbouring a TE domain (C-A-PCP-TE) are now available, i.e. SrfA-C, AB3403, EntF and ObiF1 (figure 2*a–e*) [20,28–30]. In all four crystal structures, the TE positions are dramatically different (figure 2*a-d*), suggesting that the TE domain is most probably a mobile element. Negative staining EM images of EntF showed the TE domain in various positions compared to the other domains and no density was observable for the TE domain in the corresponding crystal structure (figure 2*e*), confirming that the EntF TE domain can adopt multiple conformations [20]. The ObiF1 module has an unusual domain organization, since the TE domain is followed by a MbtH-like protein (MLP) [30]. Interestingly, in these conditions, the MLP domain anchors the TE domain to the module (figure 2*d*). These elements suggest that the high mobility of the TE domain is due to the flexibility of the short PCP-TE linker and to the fact that, in general, no successive domain imposes structural restraints on the final TE domain. As none of the structures of these four termination modules revealed the interaction between the PCP and the TE domains, it was characterized through the crystal structure of the EntF PCP-TE didomain [49]. The authors used a phosphopantetheinyl-based inhibitor loaded onto the PCP domain that stabilized the transient interaction between the PCP and TE domains [49], thus providing details of a productive PCP–TE interaction.

## 7. Non-ribosomal peptide synthetases flexibility at the supramodular scale, unrelated to the catalytic cycle

As described in the previous sections, NRPS flexibility allows the conformational rearrangements that are required for the PCP domain to interact with its catalytic partners. It is then legitimate to wonder whether NRPS flexibility is only restricted to the movements that shuttle PCP-tethered substrates to the different NRPS active sites or if there is flexibility at the supramodular scale, unrelated to the catalytic cycle. In other words, are there architectural rules governing the relationships between successive modules, or is their relationship random? In addition to the crystal structure of bimodular LgrA, structural models of multimodular NRPSs derive from low resolution techniques or from the combination of crystal structures. Indeed, in 2016, Marahiel and co-workers proposed a helical model for a hypothetical 7-module NRPS assembly by combining the C-A-PCP structure of the SrfA-C termination module (figure 2*b*) with the PCP-C cross-module structure of TycC from the tyrocidine synthetase [30,50,51]. The helical axis was occupied by the PCP domains and each module was rotated by 120° relative to the previous one.

Several EM observations indicated that NRPSs probably adopt a more flexible architecture than the helical model mentioned above. An early negative staining EM observation of a fungal 11-module NRPS, responsible for the synthesis of cyclosporin, pictured this 1.7 MDa machinery as an assembly of globular moieties, most likely modules, that could adopt either very compact or elongated structures [52]. It led to the hypothesis that NRPS modules are arranged as ‘beads on a chain’, suggesting that an NRPS assembly line would not adopt any specific architecture.

More recently, the dimodular NRPS DhbF (C_1_-A_1_-PCP_1_-C_2_-A_2_-PCP_2_ + MLP) was also observed by negative staining EM [21]. Despite the presence of AVS inhibitors that limited its conformational heterogeneity, DhbF adopted a continuum of conformations as diverse as an elongated shape, an L shape or a very compact shape. Most particles could be sorted into five classes that differed by the relative positions of the first module in relation to the second one. Therefore, although the flexibility inside a module is limited due to the stable conformation adopted by the C-A didomain, it seems that there are few limitations to the position one module can adopt relatively to the adjacent one. These data favour an irregular architecture for NRPSs; however, the fact that the number of classes is limited to five suggests that the supramodular architecture of NRPSs is not completely random. In the crystal structure of the A_1_-PCP_1_-C_2_ cross-module (figure 2*f*,*g*), there is no density for the PCP_1_-C_2_ linker, suggesting that it could be flexible [21]. Therefore, the movements of the PCP_1_-C_2_ linker combined with the absence of strong intermodule interactions could explain the various conformations adopted by the dimodular DhbF.

The six recent dimodular LgrA crystal structures provided further evidence that NRPSs do not adopt a unique stable architecture but rather a few conformations among a myriad of possibilities [22]. One striking example confirmed the flexibility of the PCP_1_-C_2_ intermodule linker. Indeed, the F_1_-A_1_-PCP_1_-C_2_-A_2_ variant was crystallized in the thiolation conformation for module 1 using a Val-AVS inhibitor and two molecules were found in the asymmetric unit (figure 2*h-j*). Within these two molecules, module 1 is identical but module 2 adopts two radically different positions. This behaviour results in two strikingly different LgrA shapes, reminiscent of the two DhbF structures observed by EM, one that is elongated and the other L-shaped. Therefore, it seems that locking one module in a specific conformation does not impose a unique conformation on the adjacent module. The most convincing evidence that NRPSs adopt a flexible architecture was obtained from the SAXS analyses of the LgrA F_1_-A_1_-PCP_1_-C_2_-A_2_ construct [22]. They indicated that the conformations adopted in the crystal structures do not exactly reflect the conformations adopted in solution. To better estimate these, Reimer and co-workers used the ensemble optimization method to generate different models that took into account flexibility parameters [53]. The ensemble generated fit very well with the experimental data, thus confirming the flexibility of LgrA. However, it cannot be excluded that LgrA flexibility is only apparent and is an effect of the absence of the other components of the assembly line. Indeed, in addition to LgrA, the linear gramicidin NRPS is composed of three other proteins [54] that could restrain the conformations that LgrA can adopt.

## 8. Concluding remarks

The structural and functional studies of NRPS multienzymes are not limited to providing details regarding the production of complex metabolites but can also be applied to the discovery of new antibiotics. Indeed, the products of these machineries are often essential for bacterial virulence, hence targeting their biosynthesis is a promising strategy to fight microbial pathogens [6]. For instance, the multi-drug resistant *Klebsiella pneumoniae* uses several siderophores for iron acquisition, including the NRPs enterobactin and yersiniabactin [55–57]. Strains deficient for yersinabactin production are much less virulent than the wild-type strains [58], suggesting that the yersiniabactin NRPS machinery could be potentially be explored as an antibacterial development target.

Moreover, the re-engineering of NRPS megaenzymes in order to produce new medically relevant molecules is of particular interest [59,60]. This prospect exists since the discovery of the modular organization of NRPSs [61]. To date, a straightforward strategy to re-engineer NRPS assembly lines to produce artificial peptides has been difficult to establish, although some successful reports of re-engineering were published [62–65]. Classical strategies using substitutions of A, C-A, PCP-C-A units or entire modules yielded only a small amount of synthesized peptide [59,60]. Recently, the Bode group successfully exploited a novel exchange strategy, using A-PCP-C exchange units (XUs) by fixing the borders of the XU within the flexible C and A domain linker [66]. They subsequently improved their strategy by dividing the C domain, placing the borders of the XU within the flexible linker that connects the N-terminal acceptor and C-terminal donor subdomains of C, yielding C_Acc_-A-PCP-C_Don_ (XUs) [67]. This strategy allowed the authors to produce very high yields of novel NRPS peptides, paving the way for new biotechnological approaches that could optimize the production of novel bioactive compounds through NRPS engineering. Therefore, an increased knowledge on the supramodular architecture of NRPSs, especially regarding the linker regions that allow enzyme flexibility, raises interesting perspectives for natural product re-engineering.

## Data accessibility

This article has no additional data.
